# SOCIAL ISOLATION AND NETWORKS DO DENSE URBAN CENTERS PROVIDE LARGE SUPPORTIVE SOCIAL NETWORK TO OLDER ADULTS? A CROSS-COUNTRY COMPARATIVE STUDY

**DOI:** 10.1093/geroni/igz038.1344

**Published:** 2019-11-08

**Authors:** Nicola Palmarini, Monica Cominato, Francesca Luppi, Margherita Ruggeri, Ivana Pais, Rosalba Schino, Mark Soto

**Affiliations:** 1 IBM Research, Cambridge, Massachusetts, United States; 2 Province of Vicenza (Public Administration), department of statistics, Vicenza, Italy, Italy; 3 Catholic University of the Sacred Hearth of Milan, Milan, Italy, Italy; 4 University of Padua, Department of Statistical Sciences, Padua, Italy, Italy; 5 Università Cattolica del Sacro Cuore, Milan, Italy, Italy; 6 University of Massachusetts Boston, Boston, Massachusetts, United States

## Abstract

The urban-rural dichotomy underpins the common approach in studying environmental conditions influencing older adults’ lives characterized by post-Second World War urban migration in both Italy and the United States (US). However, the traditional opposition urban-rural dichotomy is inadequate to study how the environmental characteristics of a geographical area can account for the heterogeneous profile of its populations and its age distribution. This study aims to overcome the traditional mobility theories as an explanatory dichotomy for understanding the distribution of the age structure of a given population. The extent of a supportive network, and the connection between the place of residence to the proximity of other residential centres can be seen as potential resources for understanding the attractiveness of certain areas for older adults. A large harmonized set of demographic and socio-economic data were collected from the Italian National Institute of Statistics (ISTAT) and the American Community Survey (ACS). An analysis at the Italian municipality- and US county-level finds the population over 75 years old are overrepresented in rural areas of both countries as would be expected by available employment opportunities, but considerable heterogeneity among both urban and rural areas exist. In particular, rural more than urban settings are based on an informal support network that is argued to rely on human proximity to produce successful aging in the community. The variation in population of older adults in rural areas, therefore, might have implications on how to achieve age-friendly communities, aside from population’s traditional mobility theories and formal support network.

